# IoT Data Quality Assessment Framework Using Adaptive Weighted Estimation Fusion

**DOI:** 10.3390/s23135993

**Published:** 2023-06-28

**Authors:** John Byabazaire, Gregory M. P. O’Hare, Rem Collier, Declan Delaney

**Affiliations:** 1 School of Computer Science, University College Dublin, D04 V1W8 Dublin, Ireland; gregory.ohare@tcd.ie (G.M.P.O.); rem.collier@ucd.ie (R.C.); 2 School of Computer Science and Statistics, Trinity College Dublin, D02 PN40 Dublin, Ireland; 3 School of Electrical and Electronic Engineering, University College Dublin, D04 V1W8 Dublin, Ireland; declan.delaney@ucd.ie

**Keywords:** data quality, internet of things (IoT), trust, big data model, data fusion

## Abstract

Timely data quality assessment has been shown to be crucial for the development of IoT-based applications. Different IoT applications’ varying data quality requirements pose a challenge, as each application requires a unique data quality process. This creates scalability issues as the number of applications increases, and it also has financial implications, as it would require a separate data pipeline for each application. To address this challenge, this paper proposes a novel approach integrating fusion methods into end-to-end data quality assessment to cater to different applications within a single data pipeline. By using real-time and historical analytics, the study investigates the effects of each fusion method on the resulting data quality score and how this can be used to support different applications. The study results, based on two real-world datasets, indicate that Kalman fusion had a higher overall mean quality score than Adaptive weighted fusion and Naïve fusion. However, Kalman fusion also had a higher computational burden on the system. The proposed solution offers a flexible and efficient approach to addressing IoT applications’ diverse data quality needs within a single data pipeline.

## 1. Introduction

The Internet of Things (IoT) has played a pivotal role in developing applications in many industries, from smart agriculture to transportation, health care, and homes. At its core, IoT is the integration of information and communication technologies into everyday processes [1]. Sensors interact with the environment and collect data, and actuators receive control signals from controllers. It is estimated that over 55.7 billion devices will be connected to the Internet, producing over 73.1 zettabytes (ZB) of data by 2025 [2]. From these IoT deployments, a vast amount of data are collected and used to advance innovations and improve decision marking.

Like many IoT solutions, however, deployments are often composed of heterogeneous sensor systems to ensure data collection [3]. The resulting data can suffer from high variability, inconsistencies, and gaps. As data are at the center of inferring new insights, it is essential to assess the quality of the data from which decisions are made. A poor understanding of the quality of the data can lead to poor decisions. Therefore, data quality assessment solutions have been proposed [4,5,6,7].

**Figure 1:**
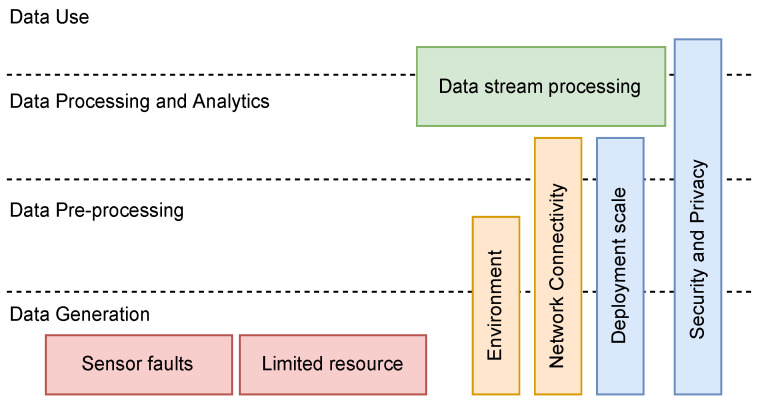
Factors that degrade IoT data across the big data model.

The factors (issues) that degrade the quality of IoT data exist at various stages throughout the big data cycle. Karkouch et al. [5] define several of these factors (sensor faults, resource limitations, network connectivity, security, and privacy) and illustrate their placement into and through the big data cycle. Figure 1 shows how various issues exist at different stages. Therefore, data quality assessment should also be carried out continuously throughout the big data cycle. Quantifying, understanding, and making these data quality issues visible throughout the big data model is essential for effective insight.

The vast opportunities IoT data presents have led to the development of many data quality assessment solutions to mitigate the effects of poor data quality. Yang et al. [8] proposed data quality assessment data based on deep learning for effective pest identification in smart agriculture. Fizza et al. [9] proposed a conceptual data quality assessment framework that is applied to monitor milk conditions for dairy farms.

More general solutions have also been proposed. Khokhlov et al. [10] proposed a framework that uses a knowledge graph to connect various DQ metrics for IoT applications. Mante et al. [11] implemented a 5D-IoT framework for heterogeneous IoT systems using the semantic descriptions of sensor observations to assess the data. Several other solutions have also been reported [12]. A common feature of all these solutions is that they all assume data quality is an isolated problem that affects a single stage of the big data cycle. Data quality challenges, however, exist at various stages throughout the big data cycle [4]. Evaluating at a single stage misrepresents the quality of the processing chain with respect to the applied data. This affects the end user applications in two ways: (1) Using data with incorrectly calculated quality assessment. (2) Lack of knowledge of where in the chain the data quality suffers, or indeed, is enhanced.

Effective data quality assessment is based on a consumer’s (application) fitness for use requirements, and indeed, this changes from one user to another [13]. Previous research [14,15] has used the phrase fitness for use and data quality interchangeably. This definition of data quality suggests that it is strongly influenced by the end user application. Each application has its own unique combination of data quality dimensions (DQD) throughout the big data cycle. This forms its fitness for use requirements. DQDs provide an acceptable way to measure data quality, for example, timeliness, accuracy, and completeness.

Existing solutions consider data quality assurance to be a necessary step at a single stage (data preprocessing) [13], applied like a *Quality Gate* with a predefined end point. Once the objective is satisfied, they assume a lasting standard of data quality [13]. It is, however, a common practice in most IoT deployments to use the same data for different applications and use cases, or enrich the data with external data sources, hence different *fitness for use* requirements. Such processes are essential for domain-specific applications [4]. As the data changes through the big data cycle, so should data quality considerations. None of the existing solutions account for the changing nature of data, and hence data quality throughout the big data cycle in a single data pipeline.

This paper implements a data quality assessment solution that uses data fusion strategies to ensure continuous end-to-end data quality assessment throughout the big data cycle. More specifically, this paper seeks to define *a tangible link between data quality, data quality stages, and their effect on the data through the stages in the big data model using data fusion*. This builds on previous research that uses trust to evaluate data quality [4,16]. This paper, therefore, uses data fusion strategies to combine quality scores at each stage into a single score, while maintaining the contribution of each stage.

Each stage has unique data quality properties, and how these are combined determines the fitness for use for different applications. To deliver this unique experience for each application *within a single data pipeline* requires a custom fusion strategy for each application. Section 2 gives a detailed motivation using two example applications: real-time analytics and historical analytics. It also highlights the importance of evaluating the computational efficiency of the different fusion strategies. To this end, the following are the core contributions of this paper:

1. Review data fusion strategies and implement a fusion engine that can integrate into big data data quality assessment frameworks for IoT applications. This paper compares three fusion strategies, Adaptive weighted fusion, Kalman’s fusion, and a Naïve fusion strategy. These are compared in terms of computational resources.
2. Using real-time analytics and historical analytics as examples, this paper illustrates how different fusion strategies can be harnessed to support different IoT applications’ unique data quality needs. Different applications can have varying data quality needs.
3. Integrate various industry standard data processing tools (e.g., Hadoop, Spark, and Kafka) to implement a real-time data quality assessment solution and also evaluate the computation resource efficiency associated with the fusion methods in an end-to-end data pipeline.

The rest of this paper is structured as follows: Section 2 highlights the need to integrate data fusion into big data quality assessment. Section 3 introduces data quality, trust, and data fusion. These are the core concepts of this paper. It also highlights related work. Section 4 outlines the theoretical and mathematical definition of the framework. Section 5 highlights the two evaluating strategies used in the paper, and finally, Section 6 gives the summary and conclusive remarks.

## 2. Motivation

This section presents the motivation for integrating data fusion strategies into end-to-end big data quality assessment for IoT applications and evaluating the computational efficiency of each fusion strategy. Data quality assessment is a complex and multidimensional process [17]. An organized methodology ensures big data quality throughout its entire lifecycle. This methodology must include a comprehensive view of quality metrics from the start of data acquisition to the implementation [18]. To this end, an end-to-end data quality assessment framework has been defined [16].

This proposed approach evaluates various data quality dimensions at different stages of the big data cycle, ultimately resulting in a single quality score that applications can easily apply. This provides visibility of the individual quality factors at each stage. IoT applications, however, have varying fitness for use requirements. Consider two broad classes of applications, real-time analytics and historical analytics, and three data quality dimensions, timeliness, accuracy, and completeness. Real-time applications require instantaneous input and fast analysis to make decisions or take actions within a specific time frame. Processing data quickly with minimal latency is essential for the development and deployment of real-time applications [19]. Some examples of this include smart intelligent transportation, fraud detection, financial trading, and many others. Real-time analytics has an essential requirement for timely data. Accuracy and completeness are considered desirable. Historical big data analytics, on the other hand, do not require timely data [19]. Some examples of it include yield prediction is smart agriculture, weather predictions, and many others. Such applications have an essential requirement for completeness. Each application has a unique fitness for use requirement and hence varying data quality requirements. Table 1 summarizes the fitness for use differences between real-time and historical analytics applications based on the above requirements.

**Figure 2:**
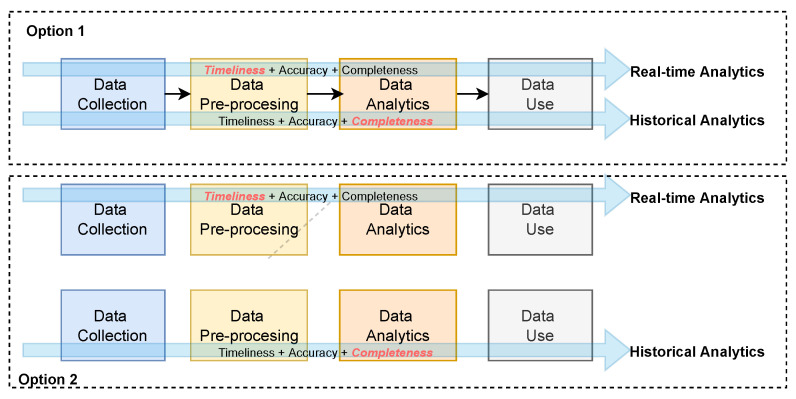
Illustrations of the different way to cater to each application’s fitness for use requirements.

A challenge therefore exists. Given a single data stream within an IoT data shared environment, how might a single data pipeline be developed that combines the various DQDs at different stages of the big data cycle to achieve a customize data quality score that uniquely satisfies each application. Currently, a few options exist: (1) A different data quality assessment process can be developed for each unique application and integrated within a single data pipeline. This is illustrated as option 1 in Figure 2. (2) Each unique application can have its data pipeline integrated with data quality assessment that meets its unique requirements. This is illustrated as option 2 in Figure 2. The first case requires maintaining two data quality assessment processes, and the last requires maintaining two data pipelines, each with its data quality assessment process. Therefore, as the number of use cases increases, so would the need to scale the pipelines. This, however, introduces redundancy, creating difficulties in consistency, and it has financial implications.

It is crucial to deliver high-quality data through a single data pipeline, but there are additional requirements that must be taken into account. Many IoT applications have the ability to off-load certain analytical processes to the lower layers of the application, for example, fog and edge [20]. Such analytical processes might require data quality assessment. A challenge exists in these kind of applications. Given various fusion strategies, when is it appropriate to choose a given strategy based on their computational resource utilization? For example, Kalman’s fusion has been reported to be computationally expensive [21], and therefore, it might not be efficient for edge applications that are characterized by low computational resources.

This paper proposes an approach that uses fusion within a single data pipeline to deliver a unique experience for each application. As the use cases increase, so might the fusion engine scale to support them. Fusion techniques have been widely used to combine different parts of the same or sometimes different systems to complement or benefit from the advantages of each. Fusion has an advantage that allows for the weighting of the combination of the DQDs to be tailored to specific applications. Therefore, this paper investigates how various fusion techniques can be integrated into big data quality assessment to deliver a single data pipeline that can uniquely cater to each application’s data quality needs. It also assesses the computational resource utilization of the fusion techniques.

## 3. Background and Related Work

This paper builds on previous research on data quality assessment using trust and compares the performance of different data fusion strategies. This section defines key concepts that form the basis for the remainder of the paper.

### 3.1. Data Quality

IoT data are a crucial part of many systems today and are used to aid decision making and create innovations. Much of the data used, however, are curated by low-cost sensors, which can be unreliable or inaccurate [22]. Assessing the quality of such data before use is therefore important. Data quality has been defined differently by several authors due to its subjective nature. Heravizadeh et al. [23] define data quality as the totality of an entity’s characteristics (data) that bear on its ability to satisfy stated and implied needs. Sidi et al. [24] define data quality as appropriateness for use or meeting user needs. This definitions aligns with the illustration given in Section 2. Not all applications have the same data quality requirements.

Lee et al. [25] present a structured method to represent and apply a wide range of metrics, possibly subjective to coefficients. This uses the concept of data quality dimensions (DQDs). DQDs provide a framework to associate wide-ranging data quality metrics to data. A DQD is a characteristic or feature of information for classifying information and data requirements [25], for example, accuracy and completeness. DQDs exist at different stages of the big data cycle. Each application has a unique weight for each DQD throughout the big data cycle. To satisfy such requirements, different fusion strategies have to be applied.

In IoT and big data, various solutions have been proposed to address the challenges of inadequate data quality. For instance, Kuemper et al. [26] introduced a framework that leverages the capabilities of IoT infrastructure and interpolation algorithms to validate crowdsourced data through sensor fusion. The authors employ machine learning techniques to validate the resulting data quality, relying on data obtained from neighboring sensors. However, a significant challenge faced by this and other data quality solutions [9,27] is the requirement for a gold standard for validation.

### 3.2. Data Fusion

The terms data and information fusion have been used interchangeably in many fields. In some, a distinction occurs, with data fusion referring to raw, unprocessed data and information fusion referring to processed data. Several definitions of data fusion exist. In a more concise definition, data fusion can be defined as a combination of multiple sources to obtain improved information; in this context, improved information means less expensive, higher-quality, or more relevant information [28]. Data fusion is a multidisciplinary area that involves several fields, and it is not easy to establish a clear and strict classification [28]. In the context of this work, since the interest is to determine the relationship between data quality properties across the big data model and how these propagate throughout the cycle, this paper adopts an approach proposed by Durrant-Whyte et al. [29]. Here, fusion is defined according to the relations between the input data sources. Three approaches are defined:

1. Complementary: The information provided by the different sources represents different parts of the system and fusing it results in a complete representation of the system.
2. Redundant: The same target is measured by two different processes, and fusing of the resulting information can lead to increased confidence.
3. Cooperative: The result of the fusion process is said to produce new information that is typically more complex than the original information.

**Figure 3:**
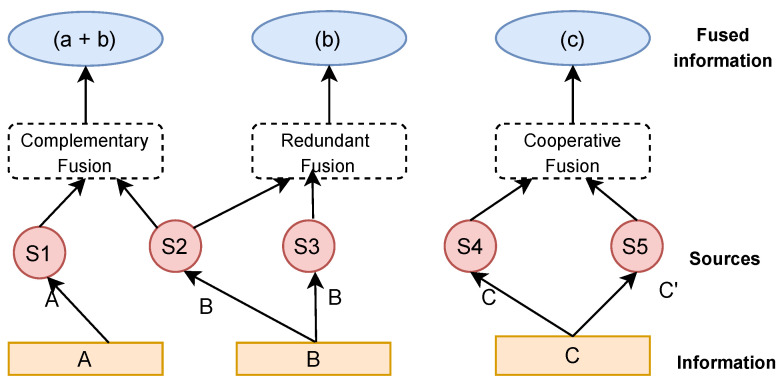
Whyte’s fusion classification based on the relations between the data sources [28].

Figure 3 summaries Whyte’s three classification strategies based on the relationships between the data sources and shows how different sources can be combined under scenarios. A more detailed description can be found here [28].

### 3.3. Related Work

The field of data fusion has grown immensely over the past years, with several techniques reported and used in many applications [30]. Traditional fusion methods such as least square estimation and arithmetic mean have been reported to have low accuracies in many situations [31,32]. Commonly used techniques such as fuzzy logic, Kalman filter, and Bayesian inference also suffer from their own limitations [33,34,35].

Kalman filter is a form of statistical interpolation that uses a model of dynamics and onboard sensor measurements to recursively determine estimates for data fusion [36]. This technique requires the system to provide the accurate state, observation equations, and prior knowledge of the statistical characteristics of the system and observation noise [30,37]. Kalman filters exist in several forms. There is the basic Kalman filter, which was designed for linear systems [38]. Other ones include the extended Kalman filter and unscented Kalman filter. Each of the preceding filters tries to mitigate the weakness of its predecessor.

Hamouda et al. [39] applied the extended Kalman filter to measure and predict agricultural parameters, including soil moisture and temperature, to filter noisy measurements in smart heterogeneous precision agriculture from energy sensor nodes deployed on a farm. Lai et al. [40] proposed a low-cost air quality monitoring and real-time prediction system based on IoT and edge computing employing a prediction algorithm that is based on a Kalman filter. This helps improve low-cost sensors’ accuracy by 27% on the edge side. Abioye et al. [41] applied a Kalman filter to a subsurface fibrous capillary irrigation system. It was used to reduce the sensor noise and help improve the accuracy of water level estimation.

The Bayesian inference uses Bayes’ formula to combine sensor data [42]. Bayes’ formula helps define the relationship between the a priori, a conditional, and a posteriori probabilities given in a hypothesis [42]. The downside of this method is that it is sensitive to prior probability distribution [33]. Razafimandimby et al. [43] applied Bayesian inference to reduce the amount of high spatiotemporal correlated data which are sent to the cloud for smart agricultural applications. Their results show a reduction in the amount of transmitted data and energy consumption, while maintaining an acceptable level of data prediction accuracy. Gevaert et al. [44] proposed a novel spectral–temporal response surfaces methodology which uses Bayesian inference to impute missing spectral information in the multispectral imagery and introduces observation uncertainties into the interpolations. This is applied to a field of potatoes for experimentation.

The fuzzy logic theory allows the uncertainty in multisensor fusion to be directly represented in the inference process [45,46,47]. When observational evidence highly conflicts with itself, however, the fusion result may be unacceptable [33]. Manjunatha et al. [48] used fuzzy logic to propose an algorithm for event detection applications in wireless sensor networks. The results show that multiple data fusion improves the reliability and accuracy of the sensed data.

Weighted fusion algorithms have gained much traction from the data fusion community [21,49,50,51]. These work by assigning a weight factor to each sensor input [21]. The core advantages include optimality, unbiasedness, and minimum mean squared error [52,53]. Moreover, compared with the Kalman filter, Bayesian estimation, and fuzzy logic theory, it can generate results without the requirement of any prior knowledge of the system or observation noises [21,49]. In the case of the proposed application, the fusion’s weighting strategy would help deliver unique fitness for use requirements for each application. Table A2 summarises the advantage and disadvantage of the various strategies.

## 4. Framework Implementation

**Figure 4:**
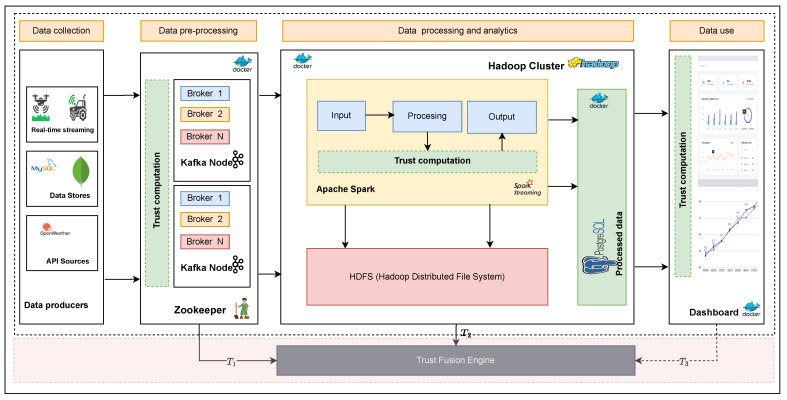
End -to-end implementation of the data pipeline with a fusion engine.

The end-to-end implementation of the system comprises two fundamental components. The first component is the data quality assessment (DQA), crucial in real-time data quality evaluation. The DQA component is responsible for assessing the data quality in real-time. It operates through three stages, as shown in Figure 4. Each has unique data quality dimensions: accuracy, completeness, consistency, and timeliness. Previous research [16] describes this in more detail.

The second core component of the implementation is the data fusion engine. This component plays a vital role in integrating and consolidating the results obtained from the three stages of the DQA. It takes the intermediate quality assessment outputs from each stage and returns a single quality score. The data fusion engine operates independently and can be configured with different fusion strategies, providing flexibility and adaptability to meet the specific needs of different applications. By combining the outputs from the DQA stages, the data fusion engine provides a holistic view of the data quality, enabling support for different application needs. Both components are modular and can apply assessments of fusion strategies independently. The following sections highlight each component in detail.

### 4.1. Data Quality Assessment (DQA)

The DQA is responsible for assessing the quality of the data from data streams before they are shared in real-time. This is based on previous research [16] that uses trust to evaluate data quality. The previous study has built and tested an end-to-end DQ assessment framework that integrates DQ assessment into the big data cycle for data-shared IoT applications [4,16]. Trust is a well-established metric that has been used to determine the validity of a piece or source of data in crowdsourced or other unreliable data collection techniques. In this paper, the terms *trust score* and *quality score* are used to mean the same thing.

Figure 4 shows the detailed internal implementation of the DQA and how it integrates with the data fusion engine to complement its functionality. The DQA is based on industry-standard data pipeline tools. A detailed description and implementation of the DQ assessment framework and the use of trust to evaluate DQ for data-shared IoT applications can be found here [4,16].

As the data stream through the framework, they are evaluated for at each stage. In Figure 4, this is represented by the *Trust computation* blocks. Each block has a set of unique DQDs used to evaluate data quality. For example, the trust block during data preprocessing ($T_{1}$) will evaluate intrinsic data quality, which in our example is timeliness. Trust evaluated during processing ($T_{2}$) evaluates investigative trust, accuracy, and completeness in our example. This computation results into a single trust score ($T_{1}$, $T_{2}$, and $T_{3}$) for each stage. $T_{3}$ is not used in this example, as it is still an element for future research. This requires defining a feedback loop from each application back into the data quality assessment framework. However, it can later be integrated into the framework without any modifications. The resulting score must be combined into a single score that represents the unique fitness for use for each end-user application.

### 4.2. Data Fusion Engine

The data fusion engine takes the output of all three trust stages and returns a single score representative of all the stages. Depending on the fusion strategy used, the resulting score should be able to satisfy the data quality requirements for each application in a homogeneous data pipeline. Fusion allows the facility to control how each stage can be weighted to achieve this objective. This paper uses an Adaptive weighted fusion strategy and compares it with other fusion strategies.

#### 4.2.1. Mathematical Formulation for Adaptive Weighted Fusion

**Figure 5:**
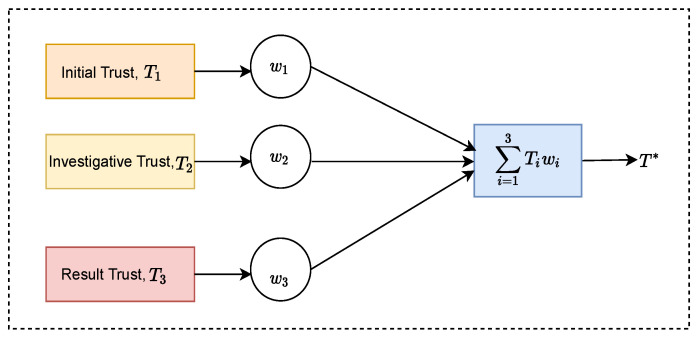
Adaptive weighted data fusion model.

Assuming there are *n* trust stages with a different trust score at each stage, the resulting weighted data fusion model is shown in Figure 5. Moreover, assume that the mean squared errors of each trust score for each stage are $\sigma_{1}^{2},\sigma_{2}^{2},\ldots ,\sigma_{n}^{2}$, and the calculated trust scores are $T_{1},T_{2},\ldots ,T_{n}$. The corresponding weight factors for the trust scores are $W_{1},W_{2},\ldots ,W_{n}$, respectively. Since each trust score is independent of the other, and all belong to the unbiased estimation of $T^{*}$, the trust score factor and weight of $T^{*}$ after fusion satisfy the following relationship:

(1) $$T^{*}=\sum_{i=1}^{n}W_{i}T_{i}\;,\;\sum_{i=1}^{n}W_{i}=1$$

Therefore, the total mean square error is

(2) $$\begin{array}{cc} \sigma^{2}= & E\left[{\left(T-\bar{T}\right)}^{2}\right]\\ = & \;E\left[{\left(\sum_{i=1}^{n}W_{i}T-\sum_{i=1}^{n}W_{i}T_{i}\right)}^{2}\right]\\ = & \;E\left[\sum_{i=1}^{n}W_{i}^{2}{\left(T-T_{i}\right)}^{2}\right]=\sum_{i=1}^{n}W_{i}^{2}\sigma_{i}^{2} \end{array}$$

Following Equation (2), the mean square error $\sigma^{2}$ is a multivariate quadratic function. Therefore, $\sigma^{2}$ must have a minimum value. Using the extreme value theory of the multivariate function, the minimum weight factor is

(3) $$W_{i}^{*}=\frac{1}{\sigma_{i}^{2}\sum_{i=1}^{n}\frac{1}{\sigma_{i}^{2}}}$$

Correspondingly, the minimum mean square error is

(4) $$\sigma_{min}^{2}=\frac{1}{\sum_{i=1}^{n}\frac{1}{\sigma_{i}^{2}}}$$

For of any number of the trust stages j (j=1,2,3,…,n), it can be concluded that

(5) $$\sigma_{min}^{2}<\sigma_{j}^{2}$$

Therefore, the overall mean square error is smaller than the mean square error of any given single stage. Moreover, the overall fused trust score will be improved compared with the trust score of a single stage. The data quality properties of each stage are reflected into the final score. Their contribution, however, is weighted depending on the fitness for use for each application. Table A1 summarises all the notations used.

#### 4.2.2. Kalman Fusion

**Figure 6:**
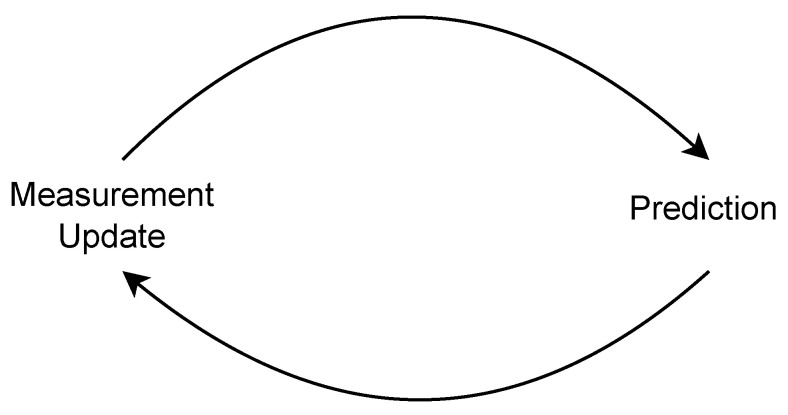
Steps of the Kalman fusion.

In 1D Kalman fusion, the system’s state and measurements are scalar values. The filter maintains two estimates of the system’s state: the predicted state, based on the previous state and the system’s dynamics, and the filtered state, based on the predicted state and the most recent measurement [54]. The Kalman fusion process utilizes these two estimates, the predicted state and the filtered state, to continuously update its understanding of the system’s state over time. As new measurements become available, the filter refines its estimates by iteratively adjusting the weights assigned to the predicted state and the measurement. This iterative update mechanism allows the Kalman filter to adapt and provide increasingly accurate state estimates as more data are assimilated. Figure 6 shows the process of measurement update and prediction.

By iterating through these equations over time, the Kalman fusion combines measurements with predictions to estimate the true state of a system. A detailed description and mathematical illustration of Kalman fusion can be found here [54].

## 5. System Evaluation

Two experiments were carried out to assess the proposed system, each serving a different purpose. The initial experiment aimed to demonstrate each fusion method’s impact on the resulting data quality score and examine how this can be used to support a specific application.

Various fusion methods employ distinct weighting schemes. For instance, when presented with two inputs, a fusion method could assign a greater weight to an input with a lower standard error and a lesser weight to one with a higher standard error. As a result, diverse fusion methods can produce varying data quality scores. Rather than incorporating numerous data quality assessment procedures into a single pipeline or constructing multiple pipelines, different fusion methods can be utilized to aid different applications by simply integrating a new fusion method or implementing a novel weighting scheme that is custom tailored to the application.

The objective of the second experiment is to evaluate and compare the computational resource consumption of the different fusion methods. While it is crucial to deliver personalized data quality scores, it is also vital to assess the impact of each fusion method on the application in terms of computational resources. Different applications perform analytics that necessitate quality assessment at varying layers. Particular layers, such as the edge, have resource constraints. This experiment, therefore, aims to facilitate improved service placement within IoT application architectures.

The experiments were conducted separately because each dataset has specific features supporting only one experiment. In the first case, a dataset with a gold standard was used to assess fitness for use by varying DQDs. In the second case, a dataset collected over a more extended period was used. Detailed descriptions of each dataset and experimental setup are provided in the following sections.

### 5.1. Dataset Description

Two datasets where used to evaluate the proposed system. Both are collected from real-world IoT deployments. The first is based on data collected in an air quality deployment. This dataset is publicly available [55]. A multisensor device was co-located with a conventional air pollution analyzer. This was used to provide the true concentration values of the target pollutants at the measurement site. These values were hence used as a gold standard. This study uses data from the CO sensor. This was collected over a one year period. This dataset is used to evaluate the fitness for use as it contains gold-standard data. This ensures that the accuracy metric can be determined and held constant, as its only a desirable DQD.

The second dataset consists of weather data collected from a real-world setup of weather stations installed across the United Kingdom between 2014 and 2020. The dataset encompasses over 100 weather stations, each recording air temperature, rainfall, relative humidity, and wind values, as well as an average of 30,000 data points yearly. This dataset serves as a test case for evaluating the system performance of the proposed fusion techniques for big sensor data systems, covering real-time and batch processing scenarios. Its deployment scale and longevity make it ideal for assessing the effectiveness of the proposed techniques.

### 5.2. Experiment 1

The goal of this experiment is to show the effects each fusion method has on the resulting data quality score and how this can be used to support various applications. The data undergo processing via the system illustrated in Figure 4. A Kafka producer reads and preprocesses the data, then sends them to Apache Spark for further processing and analytics. Preprocessing involves calculating quality score $T_{1}$, which assesses timeliness DQD, while data processing and analytics calculate the quality score, $T_{2}$, based on accuracy and completeness DQDs. These scores are sent to the fusion engine, which generates a single, usable score, as depicted in Figure 4.

To investigate varying data quality requirements, the experiment explores two scenarios by modifying essential DQDs. In the first scenario, $T_{1}$ values are varied while $T_{2}$ is held constant. $T_{1}$ values are varied by regulating the rate at which the Kafka producer transmits data to the consumer, thereby introducing delays in the data pipeline. This is expected to impact the data quality of the real-time analytics application by lowering timeliness.

The second scenario entails changing $T_{2}$ values while keeping $T_{1}$ constant, which involves introducing missing values into the dataset to impact the completeness of DQD. This manipulation can vary quality scores for $T_{2}$ depending on the rate of missing values. By virtue of the data requirements on the historical application, this manipulation should affect the overall quality score for the application.

**Figure 7:**
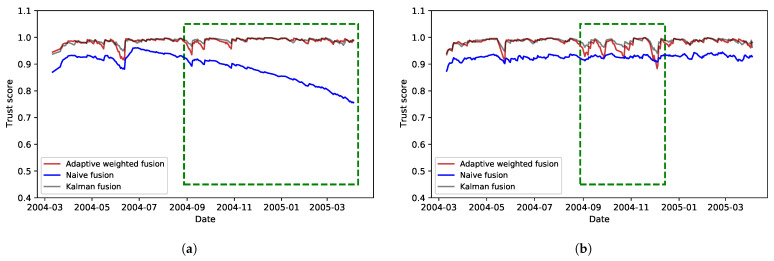
Comparing the effect of each fusion method on the resulting quality scores based on Adaptive weighted fusion, Kalman fusion, and Naïve fusion. The highlighted green areas show the effect each fusion method has on the resulting quality scores.

After calculating the scores, a distinct fusion strategy is employed for each application to enable varying weighting of the scores and produce unique, usable quality scores. As a result, two quality score curves are generated, one for each application, using a specific fusion strategy. The resulting curves for the two scenarios are shown in Figure 7a and Figure 7b, respectively.

### 5.3. Experiment 2

**Figure 8:**
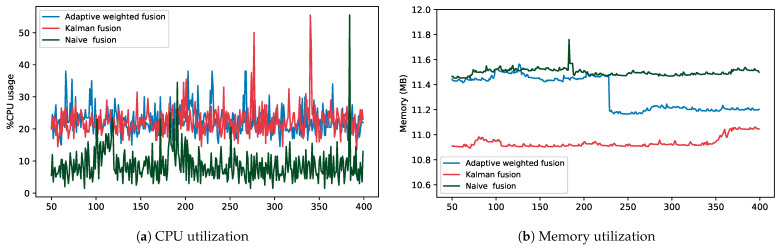
Comparing CPU and memory utilization for three fusion strategies in a real-time data streaming pipeline.

**Figure 9:**
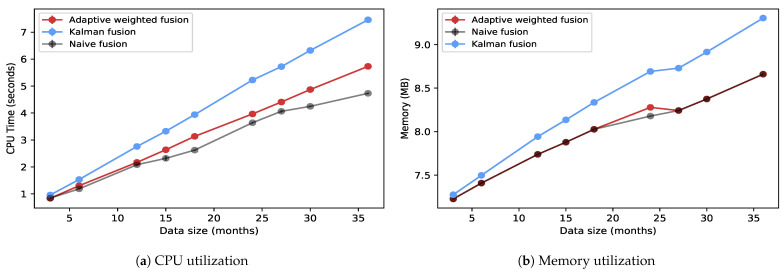
Comparing CPU and memory utilization between adaptive weighted and Kalman fusion for batch data pipeline.

This experiment evaluates the resource consumption for the various fusion methods. This is carried out for both real-time and batch processes. This measures the CPU and memory utilization. In this setup, there were no modification to the data or the DQDs. The data are processed as is. As the data are evaluated for quality, the experiment measures the CPU and memory utilization of the fusion engine. It is important to note that the results reported here are only for the fusion engine. The other parts of the system have been previously evaluated [16]. This is represented in Figure 8 and Figure 9.

### 5.4. Results and Analysis

Figure 7 shows the resulting quality scores that take into account fitness for use requirements for the two categories of applications. Figure 7a shows the resulting effect of continuously reducing timeliness and its effect on both application categories. Adaptive weighted fusion and Kalman fusion were able to maintain a more stable quality experience even when timeliness was reduced. Kalman fusion presents an overall mean quality score of 0.987 and standard deviation of 0.009. Adaptive weighted fusion presents an overall mean quality score of 0.984 and standard deviation of 0.01. Although the difference between Kalman fusion and Adaptive fusion is relatively small, the characteristics they show can be used to deliver a different quality experience to different application with varying data quality needs where timeliness is not a stringent requirement. Naïve fusion, however, was impacted by a reduction in timeliness, therefore resulting in lower data quality. It had an overall mean quality score of 0.886 and standard deviation of 0.05. In an application where timeliness is a stringent requirement, this can be useful to show the reduced data quality while taking timeliness into consideration.

Figure 7b shows results of varying completeness. The highlighted area shows the effect each fusion method has on the resulting quality scores. A reduction in $T_{2}$ did not have a significant impact on Kalman fusion. Unlike the above case, Adaptive weighted fusion presents lower quality scores at some point compared with Naïve fusion, thus showing the dynamic capacity of the fusion schemes over the Naïve approach. Overall, Kalman fusion presents a mean quality score of 0.984 and standard deviation of 0.011 compared with Adaptive weighted fusion, with a mean of 0.97 and standard deviation of 0.02. As in the above case, these differences in the resulting quality score for each fusion method can be used to deliver different quality experience to IoT applications with varying data quality needs within a single data pipeline.

As illustrated above, each fusion method had a different effect on the resulting quality score. This paper has compared only a few fusion methods and how this can help deliver unique fitness for use to two broad categories of applications. The system can easily be extended to include other fusion methods that can have custom weighting strategies to deliver data quality scores to very specific applications in IoT.

### 5.5. System Performance

System performance was evaluated for the fusion engine’s CPU time and memory utilization. These metrics have been suggested as the most appropriate when evaluating a fusion strategy [28]. The evaluation was performed for both real-time streaming and batch-processing data pipelines. It is important to note that the evaluation results reported here are only for the fusion engine. The other part of the system has been evaluated in a previous study [16].

#### 5.5.1. Real-Time Streaming

The fusion engine node is completely decoupled from the rest of the system; therefore, its resources can be fully customized depending on the workload. The fusion node was configured for the real-time streaming pipeline with 5 CPUs, each at 2.40 GHz and 32 GB of RAM. CPU utilization is measured in percentage usage, while memory is in megabytes consumed.

Figure 8a shows the comparison results for CPU utilization for three fusion strategies: Adaptive weighted fusion, Kalman fusion, and Naïve fusion for a single real-time stream job. As shown, Kalman and Adaptive weighted fusion have the highest comparable percentage of CPU utilization, with means of 22.44% and 22.54% and standard deviations of 4.39 and 4.14, respectively. Naïve fusion had the lowest values, with a mean of 8.75% and a standard deviation of 5.04. All three methods had high viability, as shown in the graph, and high and comparable standard deviation values.

Figure 8b shows the comparison results for memory utilization for the three fusion strategies. Unlike CPU utilization, Kalman fusion had the lowest values, with a mean of 10.93 MB and a standard deviation of 0.04. Kalman fusion has been suggested to be more resource-effective for smaller data sizes [56]. However, overall, all three fusion strategies had values within a close range, with means and standard deviations of 11.33 MB and 0.132 and 11.49 MB and 0.03 for Adaptive weighted and Naïve fusion, respectively. This is because, for real-time streaming jobs, a small amount of data are processed at a given time.

#### 5.5.2. Batch Processing

The system was also evaluated for batch processing workloads. The results in this section compare two fusion strategies: Adaptive weighted and Kalman fusion. They were evaluated for CPU time and memory utilization as data size increased. The data size was measured in the number of months. This was performed on a single node with 5 CPUs, each at 2.40 GHz and 32 GB of RAM.

Figure 9a,b compares CPU time and memory utilization between Adaptive weighted fusion, Kalman fusion, and Naïve fusion, respectively, as data size increases for batch processing. As shown, as data size (in months) increases, so does consumption for both CPU and memory. Adaptive weighted fusion and Naïve fusion had lower values compared with Kalman fusion. As previously reported, Kalman fusion is a highly computational fusion strategy [21].

As compared with real-time streaming, batch processing had lower memory consumption. This is partly due to the frequent memory reads and writes that streaming jobs incur. Batch processing had high CPU percentage usage compared with real-time streaming, with averages of up to 95%.

The system was also evaluated for scalability with different compute configurations. These are summarized in Table 2. The results indicate that the fusion engine could not be scaled. Increasing compute resources yielded the same average delay in CPU time and memory utilized. It should be noted, however, that this constraint is inherent in the fusion algorithms rather than the overall system. This is because, during the fusion stage, all the data have to be processed in a single stage. It should also be noted that the scalability of the DQA was reported in previous research [16]. The results show that it could scale both horizontally and vertically.

### 5.6. Discussion

This paper compares three fusion strategies which combine quality scores for a given data stream or data inputs. It can be argued that each fusion technique is suitable for a given application; that is, it presents a suitable representation of the data quality for the given application. The application of a given fusion strategy will depend on how the application views quality. For example, some applications might require consistent data quality, requiring a dynamic means to represent quality, while others might handle patches of fluctuating data quality but require overall quality above a given threshold. This can be useful to support data from sensors in difficult communication situations.

Each fusion method has a different weighting strategy which affects the final score. In Kalman fusion, higher error rates are penalized highly [52]. This can be useful in applications where a change in a single data quality dimension (for example, timeliness) should not affect the overall quality of needs of the application. Using Kalman fusion, therefore, a higher penalty would be applied to that data quality dimension to deliver the desired quality experience for that application. For example, in real-time analytics, sometimes, the data can be late; however, this should not affect the overall quality. These data can be used to calculate intermediate results, which can be updated later. Using this kind of fusion strategy, such applications can be supported.

Adaptive weighted fusion can determine the optimal weights for each source. This allows the differences between each data source’s error rate to be flexibly considered [57]. They can be useful in dynamic environments where the error rates between several data sources must be considered. This would help maintain a quality score that adapts to the error rate. This can be useful to support applications (for example, smart agriculture) that rely on data from network-constrained environments by offering a more dynamic data quality assessment experience.

Naïve fusion would assign equal weights. This can be useful in cases where we do not know the application’s needs. For scalability and resource-constrained environments, however, Adaptive weighted fusion is a better choice compared with Kalman fusion.

The goal of this work is not to imply that a given fusion method is better than the other in all general cases but rather to show that a given fusion method can deliver a better quality experience that satisfies a given application requirement.

## 6. Summary and Conclusions

This paper discussed different IoT applications’ varying data quality needs, called fitness for use. The solution integrates different fusion methods to cater to different applications’ unique data quality needs. The study investigates the impact of these fusion methods on data quality scores and their applicability in supporting diverse applications. It also evaluates the computational efficiency of the fusion methods to optimize service placement.

However, it is essential to note some limitations of the study. Firstly, the real-time and historical analytics comparison may not capture all potential scenarios and variations in data quality requirements. Different applications may exhibit distinct patterns and data characteristics, which could influence the performance of fusion methods differently. Additionally, the study focuses on only three fusion methods—Kalman fusion, Adaptive weighted fusion, and Naïve fusion—limiting the exploration of other potential fusion techniques that could enhance data quality.

In conclusion, this paper offers an insightful approach to addressing the diverse data quality needs of IoT applications through the fitness for use solution and fusion methods. However, limitations regarding the representativeness of the comparison scenarios, the limited exploration of fusion methods, and the lack of detailed resource requirements and scalability considerations should be considered when interpreting the findings and applying them in practical IoT settings. These form the basis for future work.

## Figures and Tables

**Table 1:** Fitness for use classification for real-time and historical analytics.

Application Class	Data Quality Dimensions		
	Timeliness	Accuracy	Completeness
Real-time Analytics	Essential	Desirable	Desirable
Historical Analytics	Desirable	Desirable	Essential

**Table 2:** System resource configuration for batch data pipelines.

	Conf 1	Conf 2	Conf 3	Conf 4
CPU (vcpus)	4	8	16	32
RAM (GB)	8	16	32	64

## Data Availability

The data presented in this study are available on request from the corresponding author. The data are not publicly available due to copyright.

## Appendix A

Table A1 list all the math notations used in the paper.

**Table A1:** Math notations and their definitions.

Symbol/Notation	Interpretation
σ	Mean squared error of each trust score for each stage
*T*	Trust score for each stage
$T^{*}$	Resultant trust score after fusion
*W*	Weighting factor for each stage

## Appendix B

Table A2 Summary of the advantages and disadvantages of data quality control methods and fusion approaches.

**Table A2:** Summary of the advantages and disadvantages of data quality control methods and fusion approaches.

Method	Advantages	Disadvantages
*Data Quality* *Dimensions (DQDs)*	*DQDs provide an acceptable, standardised, flexible, and measurable set of quality metrics to measure data quality.*	*They require domain knowledge to define.* *They are a nonexhaustive list which can be hard to define for IoT applications because each use case is unique.*
*Fuzzy logic*	*It is simple and capable of dealing with imprecise information and possibly including heuristic knowledge about the phenomenon under consideration* [58]	*It can produce unacceptable results if there is conflict between the input observations* [33] *It is computationally expensive* [21]
*Kalman filter*	*Kalman filter provides an unbiased and optimal estimate of a state-vectorin the sense of minimum error variance* [58]	*Requires the system to provide the accurate state, observation equations, and prior knowledge of the statistical characteristics of the system and observation noise* [30,37] *It is computationally expensive* [21]
*Bayesian inference*	*It is simple and easy to setup*	*This method is sensitive to prior probability distribution* [33]
